# CXCL10 is a novel anti‐angiogenic factor downstream of p53 in cardiomyocytes

**DOI:** 10.14814/phy2.15304

**Published:** 2022-05-11

**Authors:** Tri Wahyuni, Shota Tanaka, Ryuta Igarashi, Yoshiaki Miyake, Ayaha Yamamoto, Shota Mori, Yusuke Kametani, Masashi Tomimatsu, Shota Suzuki, Kosei Yokota, Yoshiaki Okada, Makiko Maeda, Masanori Obana, Yasushi Fujio

**Affiliations:** ^1^ 13013 Laboratory of Clinical Science and Biomedicine Graduate School of Pharmaceutical Sciences Osaka University Suita City Osaka Japan; ^2^ Laboratory of Pharmacology and Toxicology Faculty of Pharmacy Universitas Indonesia Depok City West Java Indonesia; ^3^ Laboratory of Clinical Pharmacology and Therapeutics Graduate School of Pharmaceutical Sciences Osaka University Suita City Osaka Japan; ^4^ Medical Center for Translational Research Department of Medical Innovation Osaka University Hospital Suita City Osaka Japan; ^5^ Global Center for Medical Engineering and Informatics Osaka University Suita City Osaka Japan; ^6^ Integrated Frontier Research for Medical Science Division Institute for Open and Transdisciplinary Research Initiatives Osaka University Suita City Osaka Japan; ^7^ Radioisotope Research Center Institute for Radiation Sciences Osaka University Suita City Osaka Japan

**Keywords:** angiogenesis, cardiomyocyte, CXCL10, hypoxia, p53

## Abstract

Tumor suppressor protein p53 plays crucial roles in the onset of heart failure. p53 activation results in cardiac dysfunction, at least partially by suppressing angiogenesis. Though p53 has been reported to reduce VEGF production by inhibiting hypoxia‐inducible factor, the anti‐angiogenic property of p53 remains to be fully elucidated in cardiomyocytes. To explore the molecular signals downstream of p53 that regulate vascular function, especially under normoxic conditions, DNA microarray was performed using p53‐overexpressing rat neonatal cardiomyocytes. Among genes induced by more than 2‐fold, we focused on CXCL10, an anti‐angiogenic chemokine. Real‐time PCR revealed that p53 upregulated the CXCL10 expression as well as p21, a well‐known downstream target of p53. Since p53 is known to be activated by doxorubicin (Doxo), we examined the effects of Doxo on the expression of CXCL10 and found that Doxo enhanced the CXCL10 expression, accompanied by p53 induction. Importantly, Doxo‐induced CXCL10 was abrogated by siRNA knockdown of p53, indicating that p53 activation is necessary for Doxo‐induced CXCL10. Next, we examined the effect of hypoxic condition on p53‐mediated induction of CXCL10. Interestingly, CXCL10 was induced by hypoxia and its induction was potentiated by the overexpression of p53. Finally, the conditioned media from cultured cardiomyocytes expressing p53 decreased the tube formation of endothelial cells compared with control, analyzed by angiogenesis assay. However, the inhibition of CXCR3, the receptor of CXCL10, restored the tube formation. These data indicate that CXCL10 is a novel anti‐angiogenic factor downstream of p53 in cardiomyocytes and could contribute to the suppression of vascular function by p53.

## Graphical abstract

The conditioned media from p53‐expressing cardiomyocytes decreased the tube formation of Rat Aortic Endothelial Cells. The blockade of CXCR3, a receptor of CXCL10, restored the angiogenic activity of the conditioned media from p53‐expressing cardiomyocytes.

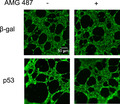

## 1 INTRODUCTION

Accumulating evidence has revealed that the interaction between cardiomyocytes and vasculature is essential for the maintenance of cardiac homeostasis. Cardiomyocytes produce angioprotective cytokines/growth factors, such as vascular endothelial growth factor (VEGF), and maintain vascular function, whereas endothelial cells prevent cardiomyocyte death by secreting cardioprotective cytokines/growth factors including neuregulin (Hemanthakumar & Kivelä, 2020; Kivelä et al., 2019; Kundumani‐Sridharan et al., 2021; Räsänen et al., 2021; Segers et al., 2018; Shen & Abe, 2021).

It is widely accepted that the myocardial induction of p53 is a critical event for the disruption of the cardiomyocyte‐vasculature interaction, leading to the onset of heart failure (Gogiraju et al., 2015; Mak et al., 2017; Men et al., 2021). Mechanistically, p53 inhibits the transcriptional activity of hypoxia‐inducible factor‐1 (HIF‐1) and suppresses the production of VEGF. Since VEGF plays important roles in cardiac homeostasis, it is reasonable that the impairment of HIF‐1/VEGF pathway by p53 results in heart failure. However, considering that HIF‐1 is activated under hypoxic condition, it is possible that p53 utilizes an alternative anti‐angiogenic signaling pathway under normoxic condition, before cardiomyocytes are exposed to hypoxia. It was hypothesized that p53 might be involved in regulation of angiogenesis via factors other than the HIF‐1/VEGF pathway under normoxia.

Chemokines are originally recognized as cytokines responsible for chemotaxis of inflammatory/immune cells. Importantly, a number of studies have revealed that some chemokines positively or negatively regulate angiogenesis (Borne et al., 2018; Eberlein et al., 2010; Noels et al., 2019; Segers & Keulenaer, 2021). For example, SDF‐1/CXCL12 functions not only as a strong chemotactic factor but also as an angiogenic factor essential for normal development. In contrast, CXCL10 exhibits anti‐angiogenic properties, though CXCL10 is also a strong chemoattractant. Intriguingly, serum concentration of CXCL10 is increased in the patients with advanced heart failure (Altara et al., 2016a; Safa et al., 2016; Wu et al., 2021), suggesting that CXCL10 production is closely associated with heart failure; however, it remains to be elucidated how CXCL10 expression is regulated in the failing heart.

In this study, we investigated whether CXCL10 expression was regulated by p53 and played a role in regulating angiogenesis as a downstream molecule of p53 in cardiomyocytes.

## 2 MATERIALS AND METHODS

### 2.1 Animals

All animal experiments conformed to the animal care guidelines of Osaka University. The experiments were conducted according to the Guide for the Care and Use of Laboratory Animals, Eighth Edition, updated by the US National Research Council Committee in 2011 and were approved by the ethics of the Committee of Osaka University and Institutional Animal Care.

### 2.2 Isolation of cardiomyocytes from neonatal rats

Neonatal rat cardiomyocytes (NRCMs) of 2‐day old Wistar rats were prepared as described previously (Wahyuni et al., 2021).

### 2.3 Doxorubicin treatment

Cardiomyocytes were seeded in 12 well plates at 1 × 10^6^ cells/well base on our preliminary experiments. Twenty‐four hours after plating, cardiomyocytes were treated with 0.01–1 μM Doxorubicin (Doxo; Kyowa Hakko, Japan) in DMEM/1%FBS for 1–24 h.

### 2.4 Overexpression of p53

Adenovirus vector expressing p53 was a generous gift from Dr. Yasuko Bando (Morimoto et al., 2011). The β‐galactosidase (β‐gal) adenovirus was generated as described previously (Iwakura et al., 2011). NRCMs were infected with the adenovirus vector expressing p53 or β‐gal, a control, at 200 multiplicities of infection (MOI) for 48 h.

### 2.5 Knockdown of p53 using siRNA

NRCMs were transfected with p53 siRNA (Sigma‐Aldrich, Assay ID; SASI_Rn01_0009‐9329; SASI_Rn01_0009‐9330; SASI_Rn01_0009‐9332) or control siRNA (Sigma‐Aldrich SIC002‐10NMOL) at a final concentration of 20 nM using Lipofectamin RNAiMAX (Thermo Fisher Scientific, #13778030) for 48 h and stimulated with Doxo for 24 h.

### 2.6 Cell viability assay

NRCMs were overexpressed or knocked down for p53, as described above. Cell viability was evaluated using the CellTiter Blue assay kit (Promega) according to the manufacturer`s protocol. The fluorescence was measured at 560/590 nm with SpectraMAX M5e (Molecular Device).

### 2.7 Microarray analysis

Total RNA was prepared from NRCMs overexpressing p53 or β‐gal, a control. The comprehensive expression comparison was performed by Rat Gene Chip Gene 2.0ST Array, Transcriptome Viewer, PANTHER‐Gene List Analysis (GO) GENE ONTOLOGY Heatmapper (http://www.heatmapper.ca/).

### 2.8 Reverse transcription and quantitative PCR

Total RNA and cDNA were prepared as described previously (Wahyuni et al., 2021). The mRNA level of p53, CXCL10, p21, and VEGF was normalized to that of GAPDH.

The sequences of the primers used in this study are as follows: Rat p53 forward, CCTCTGTCATCTTCCGTCCCT; reverse, AGCTGGCAGAACAGCTTATTGAG, Rat CXCL10forward, TGCAAGTCTATCCTGTCCGC; reverse, CTCTCTGCTGTCCATCGGTC, Rat p21 forward, GTCTTGCACTCTGGTGTCTG; reverse, CTGCGCTTGGAGTGATAGAAATC, Rat VEGF forward, CAGAAAGCCCATGAAGTGGTGAA; reverse, AAGATGTCCACCAGGGTCTCAAT, Rat GAPDH forward, CATCACCATCTTCCAGGAGCG; reverse, GAGGGGCCATCCACAGTCTTC.

### 2.9 Enzyme‐Linked Immunosorbent Assay (ELISA)

CXCL10 protein level in conditioned media, secreted from cardiomyocytes, was assessed by Rat IP‐10/CXCL10 ELISA Kit (Elabscience, #E‐EL‐R0546) according to the manufacturer's protocol. The absorbance (450 nm) was detected with EMax Plus (Molecular Devices).

### 2.10 Western blot

Western blotting was performed as described previously (Yamamoto et al., 2021). Anti‐p53 (1:1,000, #2524, Cell Signaling Technology) and anti‐GAPDH (1:1,000, #MAB374, Millipore) antibodies were used as primary antibodies. HRP‐conjugated goat‐anti mouse IgG was used as a secondary antibody. The protein bands were detected using chemiluminescence reagent (Chemi‐Lumi One Super, Nacalai tesque, Japan; ECL Western blotting substrate, Promega, USA) and LAS 4010 Imaging system (GE Healthcare). Protein band intensity was determined using NIH ImageJ software and is normalized with GAPDH signal intensity.

### 2.11 Rat Aortic Endothelial Cell (RAOEC) culture

Rat Aortic Endothelial Cells (RAOEC) was purchased from Cell Applications, Inc. (San Diego, CA, USA, Cat.# R304‐05a). The cells were cultured according to the user manual and grown in Attachment Factor Solution (AFS) (Cell Applications, Inc. Cat.# 123‐100)‐coated 100 mm tissue culture dish containing rat endothelial cell growth media (Cell Applications, Inc. Cat.# R211K‐500) in a humified incubator at 37°C in 5% CO_2_. The cells were plated and allowed to reach 80% confluency. The cells were used between passages three and eight.

### 2.12 Preparation of conditioned media

NRCMs were transfected with the adenovirus vector expressing p53 or β‐gal for 48 h. After changing to the endothelial cell growth medium, the cells were cultured for 48 h, and the conditioned medium was collected and filtered. The conditioned medium was used to treat a new batch of RAOEC.

### 2.13 Endothelial cell tube‐formation assay

The angiogenesis assay was performed using RAOEC and the tube formation assay kit (Abcam, #ab204726), according to the manufacturer's protocol. After coating the wells with the extracellular matrix, 5 × 10^5^ RAOECs were seeded to each well and cultured with the conditioned media contained AMG 487 (MedChemExpress), a selective antagonist of CXCR3. The cells were incubated for 6–8 h at 37°C with 5% CO_2_ and stained according to the manufacturer's instructions. The formation of vessels‐like structures was examined and the images were taken by fluorescence microscope BZX710 (KEYENCE). The number of tube formations was counted by a researcher who was blind to the assay condition, as a previous report (Hyun et al., 2021; Li et al., 2020).

### 2.14 Statistical analysis

All data are expressed as the mean ± SD. Statistical analyses were performed by Statcel Ver.4 (The Publisher OMS). Comparisons between the two groups were conducted by student's *t*‐test. Multiple Comparisons were conducted by one‐way ANOVA followed by Tukey–Kramer test or Dunnett test. *p < 0.05 and **p < 0.01 were considered to be statistically significant.

## 3 RESULTS

### 3.1 The overexpression of p53 upregulated the expression of CXCL10 in cultured cardiomyocytes

**FIGURE 1:**
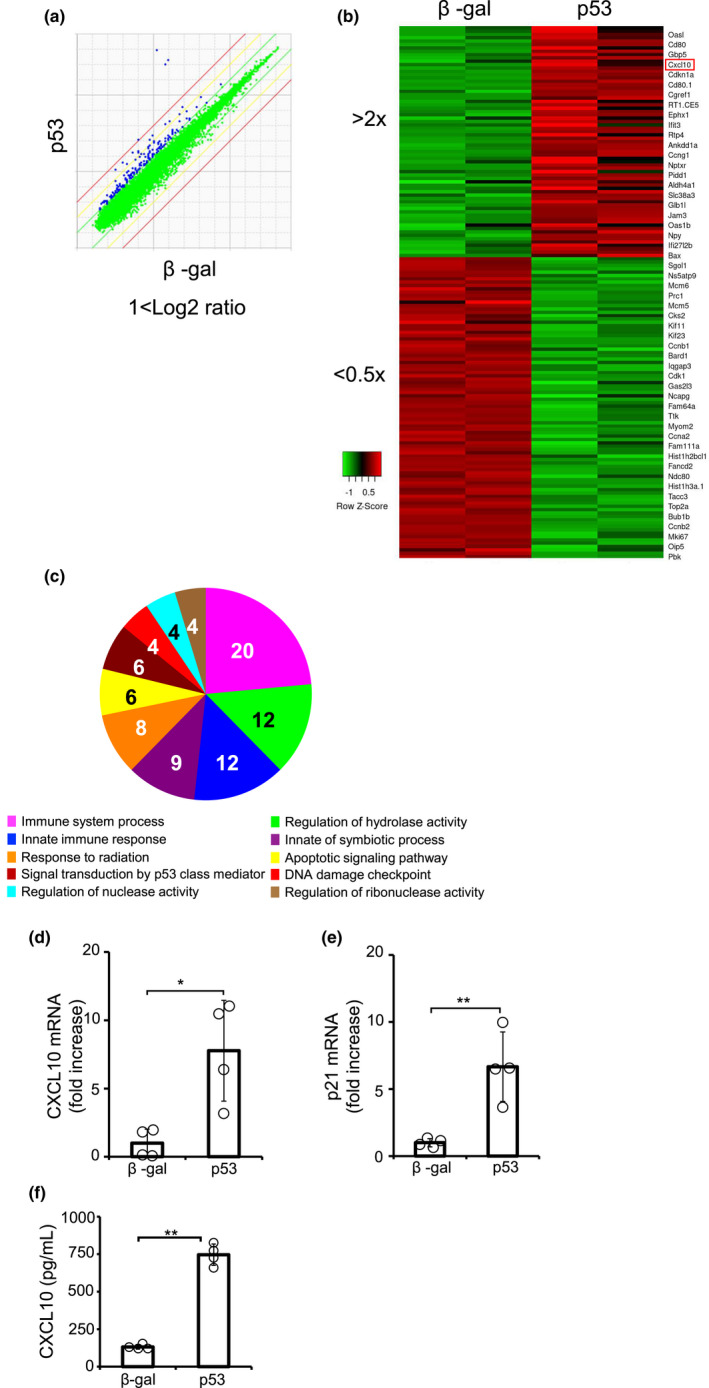
p53 induced CXCL10 expression in cardiomyocytes. DNA microarray was performed using p53‐overexpressing NRCMs. (a) Scatter plot shows the genes colored blue with >2‐fold upregulate in p53‐overexpressing NRCMs compared with β‐gal. (b) Heat map shows genes colored red with >2‐fold change and green with <0.5‐fold change between β‐gal and p53. (c) Gene ontology analysis. (d, e) The expression of (d) CXCL10 and (e) p21 transcripts was measured by qPCR (*n* = 4). Experiments were performed three times with similar results. (f) The protein expression of CXCL10 in culture media of p53‐overexpressing NRCMs was measured by ELISA (*n* = 3). *p < 0.05, **p < 0.01. by Student's *t*‐test.

It is widely accepted that p53 plays an important role in the pathogenesis of heart failure, however, the molecular mechanisms remain to be fully elucidated. To explore the downstream targets of p53 signaling in cultured cardiomyocytes, total RNA was prepared from cardiomyocytes transfected with an adenoviral vector expressing p53 or β‐gal, a control, for 48 h, and DNA array analysis was performed. The Scatter plot was shown (Figure 1a). The heatmap for gene expression data demonstrated that 23 gene transcripts were induced by p53 more than 2‐fold and that these transcripts included p21 (Cdkn1a) and Bax, well‐known targets downstream of p53 (Figure 1b). To explore the biological functions of the genes whose expression was regulated by p53, the PANTHER classification system was used (Figure 1c) (Thomas et al., 2003). As a result, genes involved in “immune system process” were the most frequently altered. Among the 23 genes induced by p53, we focused on CXCL10 because CXCL10 participates in immune regulation as a chemokine. Moreover, CXCL10 modulates the biological functions of endothelial cells as an anti‐angiogenic factor and is associated with the progression of heart failure (Borne et al., 2018; Saxena et al., 2014). Consistent with microarray analysis, quantitative RT‐PCR confirmed that CXCL10 expression was significantly upregulated by the transfection of p53, as is the case with p21 (Figure 1d,e). Significantly, ELISA assay revealed that the protein expression of CXCL10 was increased in the culture media of NRCMs overexpressing p53, suggesting that p53‐induced CXCL10 was secreted from NRCMs (Figure 1f). In addition, overexpression of p53 decreased cell viability in cardiomyocytes compared with control (Figure S1a). Taken together, CXCL10 was induced by p53 in cardiomyocytes.

### 3.2 Doxorubicin induced CXCL10 expression in cardiomyocytes through increased p53

**FIGURE 2:**
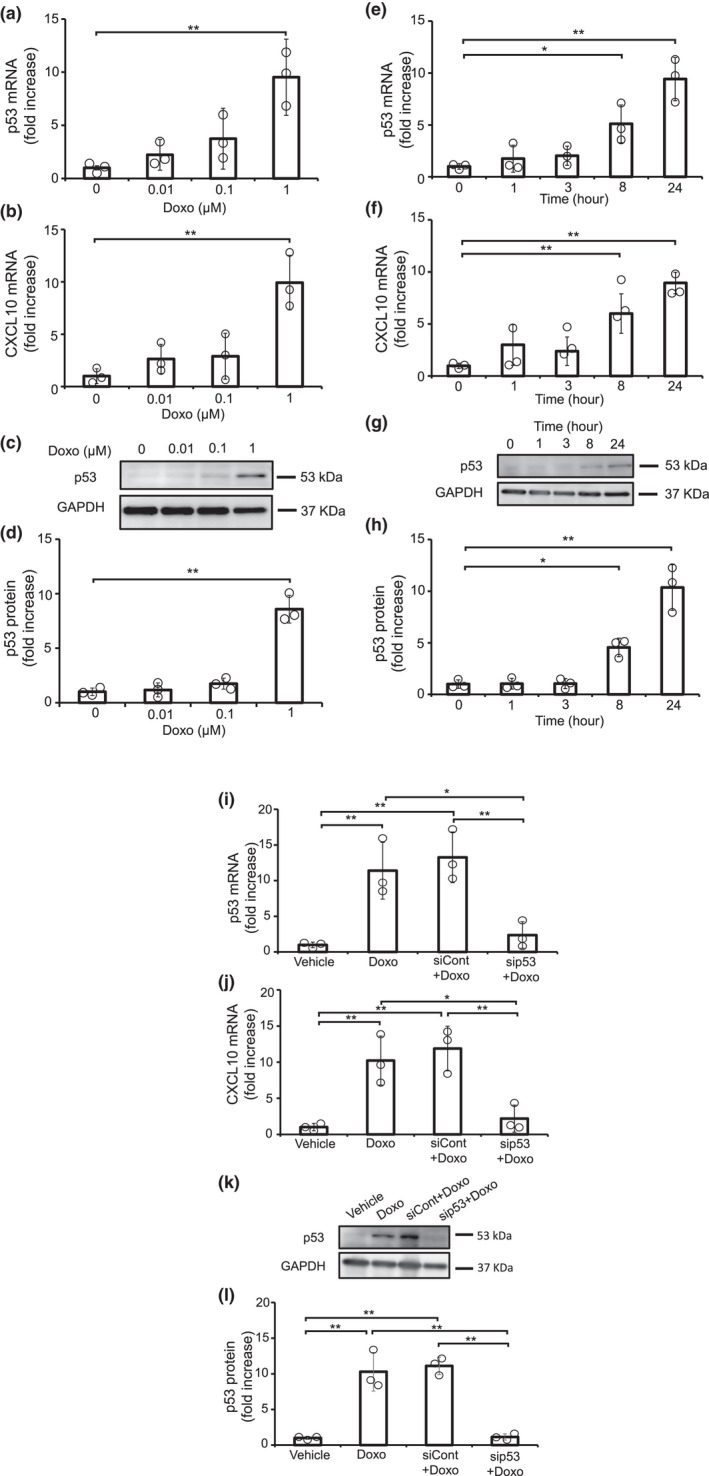
Doxorubicin induced the expression of CXCL10 in cardiomyocytes through p53 in cardiomyocytes. (a–h) NRCMs were stimulated with Doxo for indicated concentration and time. (i–l) NRCMs were transfected with siRNA for p53 (sip53) or control (siCon) for 48 h, followed by stimulation with Doxo for 24 h. The expression of p53 (a, e, i) and CXCL10 (b, f, j) mRNA was measured by qPCR. (*n* = 3). (c, g, k) The expression of p53 protein was analyzed by immunoblotting. Representative data are shown. (d, h, l) The band intensities of p53 were measured (*n* = 3). Experiments were performed three times with similar results. (a–h). *p < 0.05, **p < 0.01 by one‐way ANOVA followed by Dunnett test. (i–l) *p < 0.05, **p < 0.01 by one‐way ANOVA followed by Tukey–Kramer test.

Doxo, a chemotherapeutic drug, induces cardiac toxicity. Since Doxo upregulates the expression of p53 (Liu et al., 2008; McSweeney et al., 2019), we examined whether Doxo induces CXCL10 in cardiomyocytes through p53. NRCMs were stimulated with various concentrations of Doxo for 24 h, and the expression of p53 and CXCL10 mRNA was analyzed by quantitative PCR (Figure 2a,b). As a result, both p53 and CXCL10 were significantly induced by Doxo. Consistently, Doxo significantly increased the protein level of p53 (Figure 2c,d). Next, we addressed the time dependency of Doxo‐induced expression of p53 and CXCL10. The significant increase of p53 and CXCL10 was observed at 8 h and 24 h after Doxo stimulation (Figure 2e–h).

To make clear the causality between p53 activation and CXCL10 induction in Doxo‐treated cardiomyocytes, p53 gene was knocked down by using siRNA (Figure 2i–l). We confirmed that the knockdown of p53 did not affect cell viability in cardiomyocytes (Figure S1b). The knockdown of p53 suppressed Doxo‐induced expression of CXCL10, indicating that the activation of p53 is essential for Doxo‐induced upregulation of CXCL10.

### 3.3 Hypoxia synergistically elevated CXCL10 expression with p53

**FIGURE 3:**
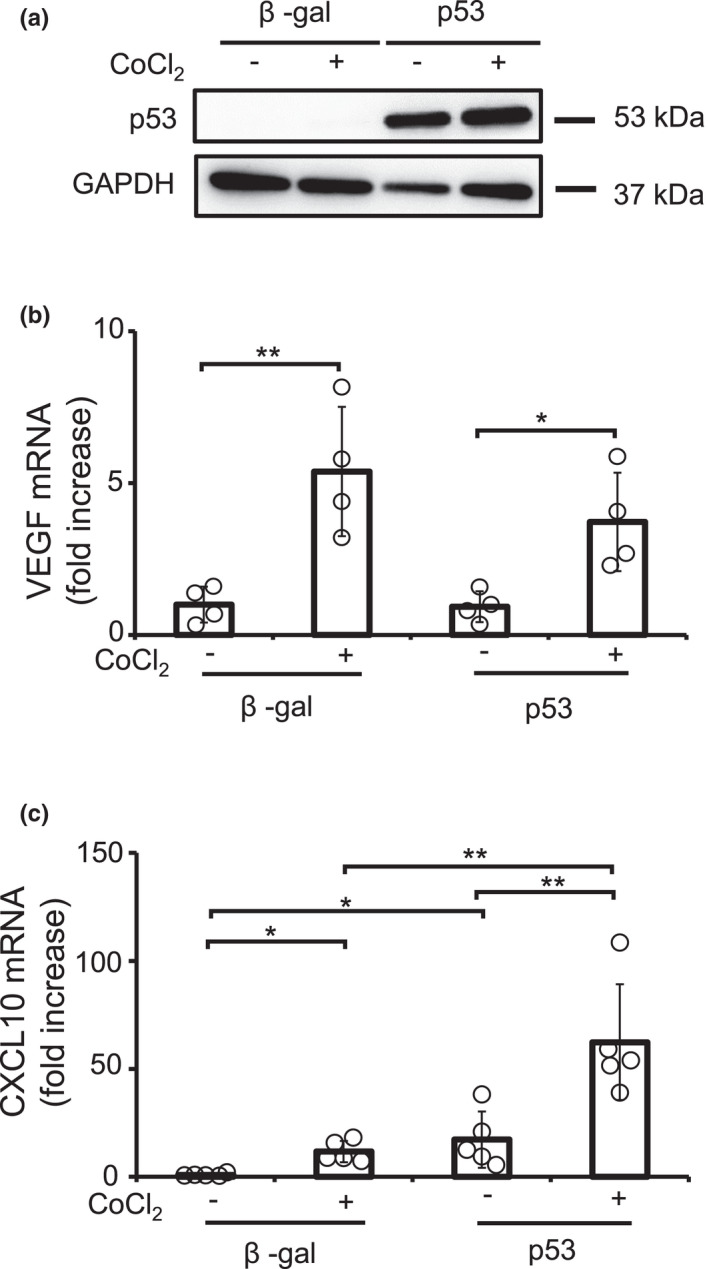
Hypoxia synergistically elevated the expression of CXCL10. p53‐overexpresisng NRCMs were treated with CoCl2 for 24 h. (a) The expression of p53 and GAPDH protein was analyzed by immunoblotting. Representative data were shown. The expression of (b) VEGF and (c) CXCL10 transcripts was measured by qPCR (*n* = 4). Experiments were performed three times with similar results. *p < 0.05, **p < 0.01 by one‐way ANOVA followed by Tukey–Kramer test.

Previously, it was reported that p53 inhibited the function of HIF‐1, leading to the downregulation of VEGF. Therefore, we examined the effects of hypoxia on p53/CXCL10 signaling pathway (Figure 3). Hypoxic stress was induced by the treatment with CoCl_2_ (Li et al., 2018; Lopez‐Sanchez et al., 2014). The CoCl_2_ treatment‐induced VEGF expression in cardiomyocytes expressing β‐gal, while VFGF expression had a tendency to be suppressed by the overexpression of p53 under hypoxic condition, but not significant. As described previously, hypoxic stress significantly increased CXCL10 expression in β‐gal‐expressing cardiomyocytes. Importantly, p53‐mediated CXCL10 induction was remarkably enhanced by CoCl_2_ treatment, indicating that p53/CXCL10 signaling pathway is enhanced by hypoxia.

### 3.4 The conditioned media from p53‐expressing cardiomyocytes suppressed the tube formation of endothelial cells in a CXCR3‐dependent manner

**FIGURE 4:**
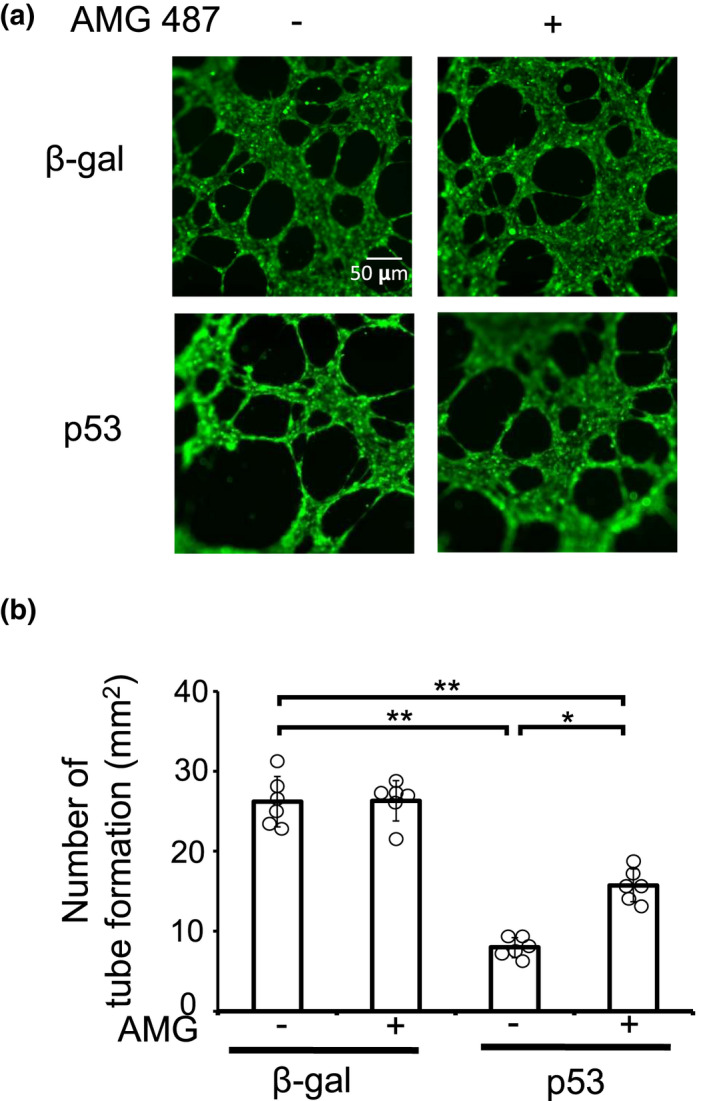
p53‐induced CXCL10, secreted from cardiomyocytes, inhibited the tube formation of endothelial cells. Culture media were collected from p53‐ or β‐gal‐overexpressing NRCMs. RAOECs were cultured with the conditioned media in the presence of AMG487, an inhibitor of CXCR3. Angiogenesis assay was performed. (a) Representative images were shown. (b) The number of tube formation was measured (*n* = 6). *p < 0.05, **p < 0.01 by one‐way ANOVA followed by Tukey–Kramer test.

CXCL10 was the only chemokine that utilizes CXCR3 as a receptor and has been reported to have anti‐angiogenic properties. Thus, to examine the angiogenic activities of CXCL10 secreted from cardiomyocytes, RAOECs, a rat endothelial cell line, were cultured with the conditioned media from cardiomyocytes expressing β‐gal or p53 in the presence of various concentrations of AMG487, an inhibitor of CXCR3 (Figure 4). Angiogenesis assay demonstrated that the conditioned media from p53‐expressing cardiomyocytes decreased the tube formation of RAOECs compared with those from β‐gal. Importantly, the blockade of CXCR3 restored the angiogenic activity of the conditioned media from p53‐expressing cardiomyocytes, suggesting that the activation of p53 signaling in cardiomyocytes controls vasculature formation via CXCL10/CXCR3 axis.

## 4 DISCUSSION

In this study, we have demonstrated that tumor suppressor protein p53 induces CXCL10, an anti‐angiogenic chemokine, in cardiomyocytes. The adenoviral overexpression of p53 upregulated the expression of CXCL10 in cultured cardiomyocytes. The treatment with Doxo induced the CXCL10 through the increase of p53. Interestingly, hypoxia enhanced the p53‐mediated expression of CXCL10. Finally, the conditioned media from p53‐expressing cardiomyocytes exhibited the anti‐angiogenic activity compared with control and this suppressive effect was abrogated by the blockade of CXCR3, the receptor of CXCL10. These data propose that p53/CXCL10 axis could be a novel anti‐angiogenic pathway in cardiomyocytes.

The myocardial induction of p53 is a critical event for the transition from compensatory to decompensatory status in the onset of heart failure. Mechanistically, the inhibition of angiogenesis by p53 is thought to be important in the process. Previously, it was reported that p53 suppressed the biological function of HIF‐1 and inhibited the production of VEGF, leading to the suppression of angiogenesis. Here, we identified CXCL10 as a novel anti‐angiogenic factor downstream of p53 in cardiomyocytes. Interestingly, p53‐mediated induction of CXCL10 was enhanced by hypoxia, suggesting that p53‐expressing cardiomyocytes reduced the angiogenic activity in response to hypoxic stress. Considering that cardiomyocyte hypertrophy is associated with hypoxia and that myocardial hypertrophy precedes heart failure, our findings support the concept that the induction of p53 is a trigger of the onset of heart failure.

A number of studies have demonstrated that Doxo upregulates the expression of p53 and induces apoptosis in cardiomyocytes, leading to heart failure (Liu et al., 2008; McSweeney et al., 2019). In fact, p53 overexpression decreased the viability of cardiomyocytes (Figure S1a). However, we treated cardiomyocytes with Doxo in the presence of FBS and found that Doxo treatment enhanced CXCL10 expression without inducing cardiomyocyte apoptosis. Therefore, p53 differentially activates the apoptotic signaling pathway and anti‐angiogenic pathway in cardiomyocytes. Further studies would be required to evaluate the clinical significance of the antiangiogenic effects of p53 in Doxo‐induced cardiotoxicity.

Recently, it was demonstrated that CXCL10 is a circulating inflammatory marker in patients with advanced heart failure (Altara et al., 2016b). Similarly, it is also reported that circulating levels of CXCL10 is associated with heart failure (Leavitt et al., 2020). These clinical findings demonstrate that blood concentration of CXCL10 is elevated in patients with heart failure; however, the mechanisms of CXCL10 induction remain to be addressed. Our data presented here propose the possibility that CXCL10 could be produced from failing hearts that express p53.

A limitation of this study is that the pathophysiological significance of p53/CXCL10 axis remains to be fully addressed because the expression of CXCL10 is regulated both in p53‐dependent and independent manner *in vivo*. Based on the data from *in vitro* angiogenesis assay, CXCL10, produced from p53‐expressing cardiomyocytes, is likely to contribute to the suppression of tube formation of endothelial cells.

In conclusion, we demonstrated that p53 induces CXCL10 in cardiomyocytes for the first time. These data propose that p53/CXCL10 axis could contribute to the onset and/or progression of heart failure.

## ETHICS STATEMENT

Study protocols of animals were approved by the ethics of the Committee of Osaka University and Institutional Animal Care (approval number: Douyaku 28‐14, R03‐16). Genetic modification experiments were conducted in accordance with the regulations for genetic modification experiments at Osaka University (approval number: (I) 04809).

## AUTHORS’ CONTRIBUTION

Conceived and designed research: Tri Wahyuni, Ryuta Igarashi, Shota Tanaka, Masanori Obana, Yasushi Fujio. Performed experiments: Tri Wahyuni, Ryuta Igarashi, Shota Tanaka, Yoshiaki Miyake, Ayaha Yamamoto, Shota Mori, Yusuke Kametani, Masashi Tomimatsu. Analyzed data: Tri Wahyuni, Shota Tanaka, Shota Suzuki, Kosei Yokota. Interpreted results of experiments: Tri Wahyuni, Shota Tanaka, Ryuta Igarashi, Yoshiaki Okada, Makiko Maeda, Masanori Obana, Yasushi Fujio. Prepared figures: Tri Wahyuni, Shota Tanaka, Ryuta Igarashi. Drafted manuscript: Tri Wahyuni, Ryuta Igarashi, Yasushi Fujio. Edited and revised manuscript: Shota Tanaka, Masanori Obana, Yasushi Fujio. The approved final version of the manuscript: all.

## Supporting information



Fig S1 and unedited western blot images
