# Supplementary material for: CXCL10 is a novel anti‐angiogenic factor downstream of p53 in cardiomyocytes
Source: Physiol Rep. 2022 May 11;10(9):e15304. doi: 10.14814/phy2.15304 (PMC9091994; doi:10.14814/phy2.15304)

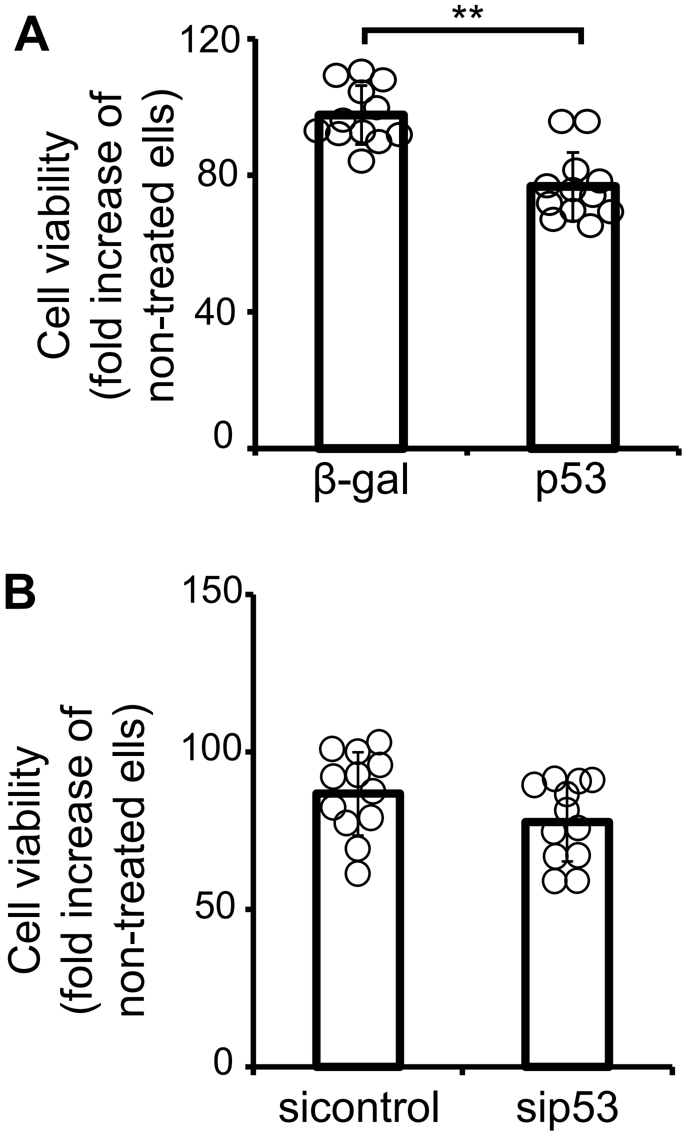

Supplementary Figure 1

Unedited western blot images

Figure 2C

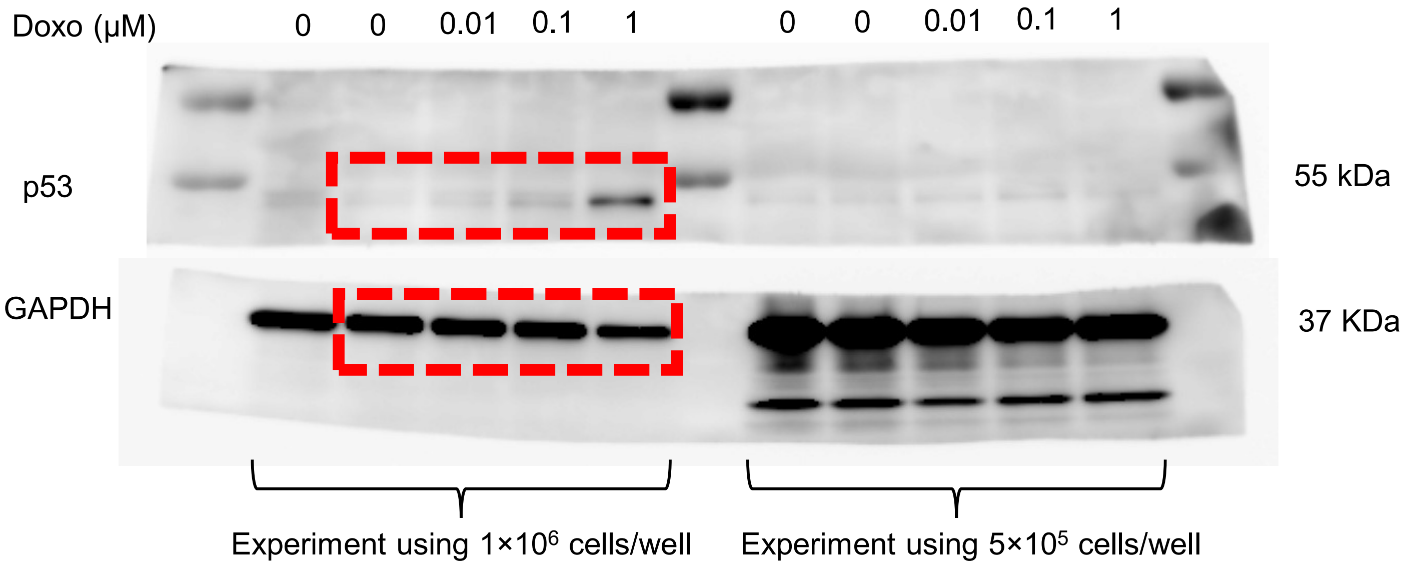

Unedited western blot images

Figure 2G

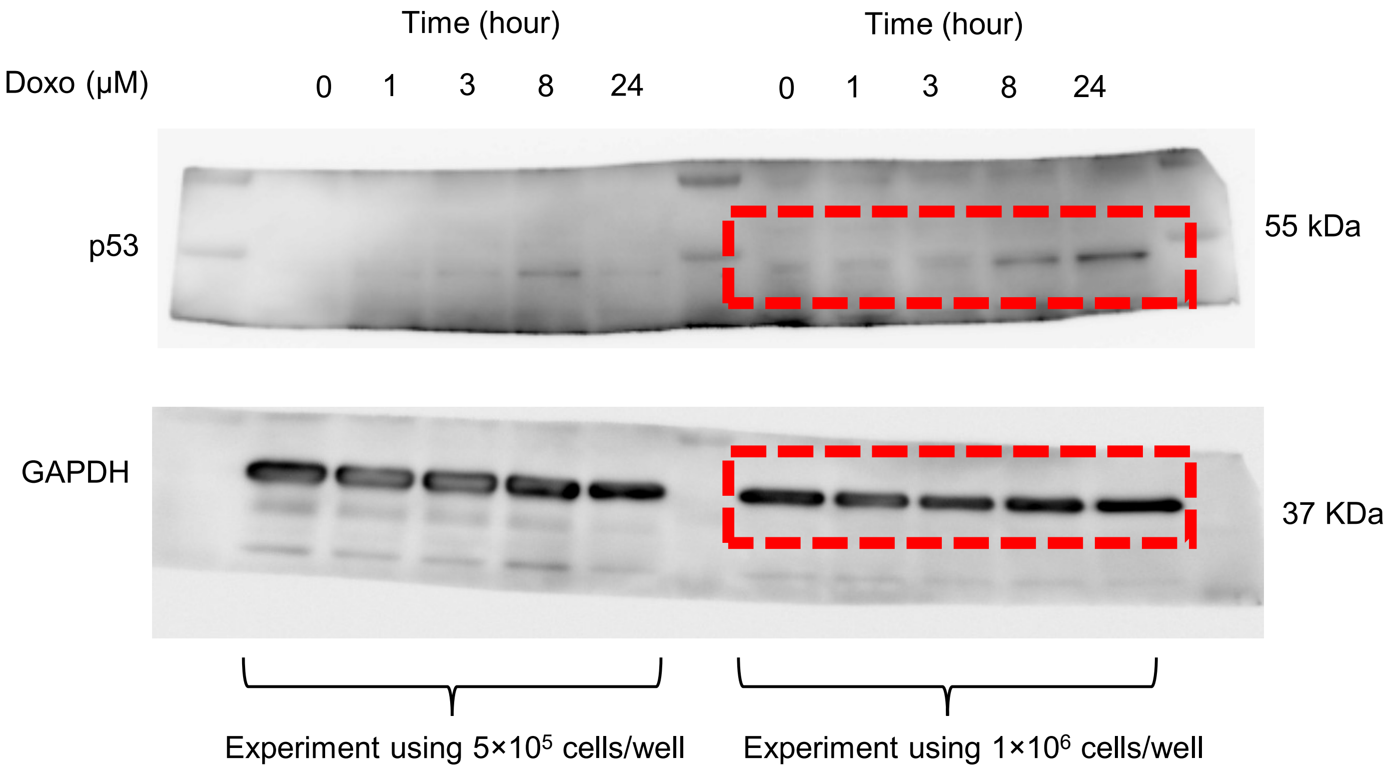

Unedited western blot images

Figure 2K

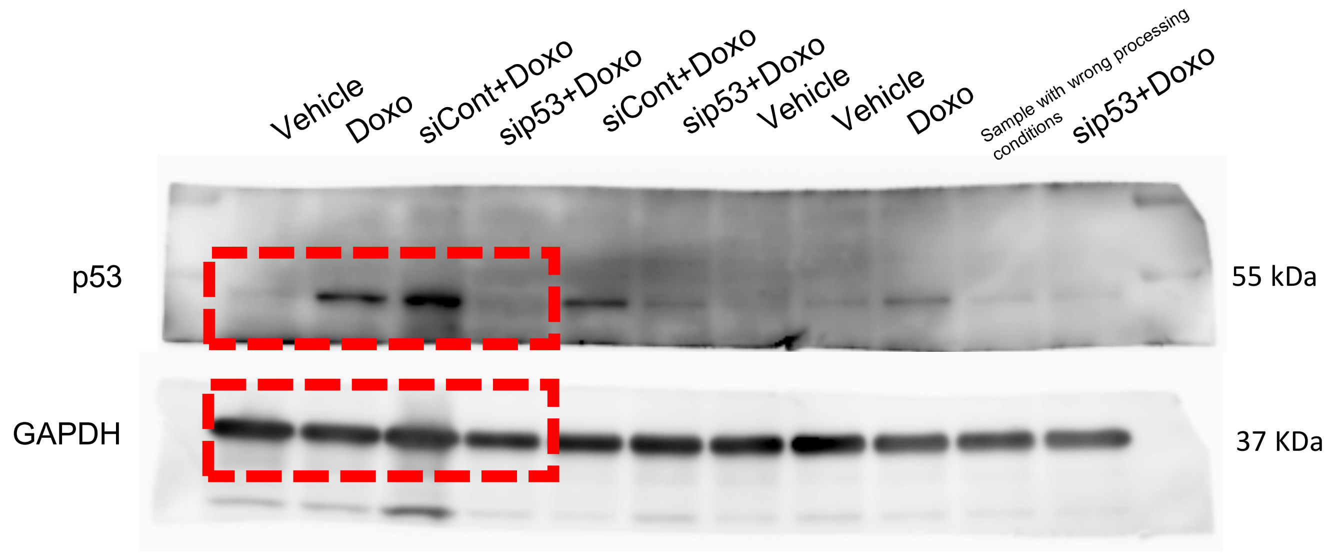

Unedited western blot images

Figure 3A

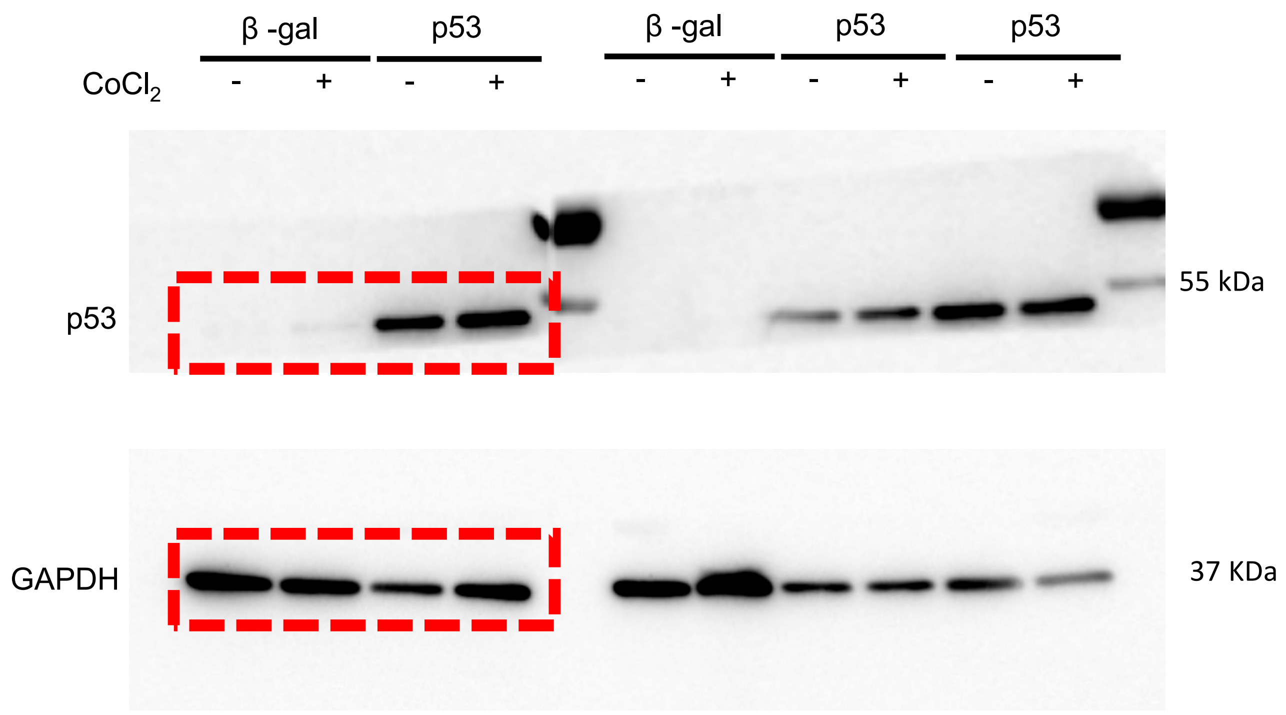

Supplement: Supplementary file 1 — Fig S1 and unedited western blot images [file PHY2-10-e15304-s001.pdf]
